# Differentiated neuroblastoma cells remain epigenetically poised for de-differentiation to an immature state

**DOI:** 10.1242/dmm.049754

**Published:** 2023-12-27

**Authors:** Richard A. Guyer, Nicole Picard, Jessica L. Mueller, Kensuke Ohishi, Abigail Leavitt, Andrew J. Murphy, Kristine M. Cornejo, Ryo Hotta, Allan M. Goldstein

**Affiliations:** ^1^ Department of Pediatric Surgery, Massachusetts General Hospital, Boston, MA 02114, USA; ^2^ Drug Discovery Laboratory, Wakunaga Pharmaceutical Co. Ltd., Akitakata, Hiroshima 739-1195, Japan; ^3^ Department of Surgery, St. Jude Children's Research Hospital, Memphis, TN 38015, USA; ^4^ Department of Pathology, Massachusetts General Hospital, Boston, MA 02114, USA

**Keywords:** CD49b, Enhancers, *Itga2*, Neural crest, Neuroblastoma, Super enhancers

## Abstract

Neuroblastoma is the most common extracranial solid tumor of childhood and accounts for a significant share of childhood cancer deaths. Prior studies utilizing RNA sequencing of bulk tumor populations showed two predominant cell states characterized by high and low expression of neuronal genes. Although cells respond to treatment by altering their gene expression, it is unclear whether this reflects shifting balances of distinct subpopulations or plasticity of individual cells. Using mouse and human neuroblastoma cell lines lacking *MYCN* amplification, we show that the antigen CD49b (also known as ITGA2) distinguishes these subpopulations. CD49b expression marked proliferative cells with an immature gene expression program, whereas CD49b-negative cells expressed differentiated neuronal marker genes and were non-cycling. Sorted populations spontaneously switched between CD49b expression states in culture, and CD49b-negative cells could generate rapidly growing, CD49b-positive tumors in mice. Although treatment with the chemotherapy drug doxorubicin selectively killed CD49b-positive cells in culture, the CD49b-positive population recovered when treatment was withdrawn. We profiled histone 3 (H3) lysine 27 acetylation (H3K27ac) to identify enhancers and super enhancers that were specifically active in each population and found that CD49b-negative cells maintained the priming H3 lysine 4 methylation (H3K4me1) mark at elements that were active in cells with high expression of CD49b. Improper maintenance of primed enhancer elements might thus underlie cellular plasticity in neuroblastoma, representing potential therapeutic targets for this lethal tumor.

## Teaser

**Summary:** Neuroblastoma cells lacking *MYCN* amplification exhibit distinct subpopulations associated with differential gene expression programs and can interconvert between a state characterized by expression of neuronal genes and a de-differentiated state.

## INTRODUCTION

Neuroblastoma is the most common extracranial solid tumor in children and accounts for 15% of pediatric cancer deaths annually (Newman et al., 2019). These tumors arise when normal differentiation of neural crest cells into sympathetic neurons of the peripheral nervous system is disrupted (Kildisiute et al., 2021). The disease is stratified based on clinical and molecular characteristics, with high-risk tumors carrying a dismal prognosis (Cohn et al., 2009). Although *MYCN* amplification is the most common mutation found in high-risk lesions, over half of these tumors do not display *MYCN* amplification (Lee et al., 2018; Yanishevski et al., 2020).

Cellular identity is defined by the proteins that have been translated at any moment, and protein translation requires transcription of genomic information into RNA. RNA expression is determined by the enhancer elements active in a given cell (Long et al., 2016). Primed enhancers, marked by monomethylation or dimethylation of lysine 4 on histone 3 (H3K4me1/2), are not actively engaged in promoting transcription, whereas active enhancers, denoted by acetylation on lysine 27 on histone 3 (H3K27ac), are bound by transcription factors and replication machinery (Creyghton et al., 2010). Stem and progenitor cells are characterized by a broad repertoire of primed enhancers that can be activated to trigger a change in transcriptional state (Crispatzu et al., 2021; Rada-Iglesias et al., 2011). As cells progress through differentiation options, enhancers are decommissioned via loss of H3K4me1/2 marks to limit fate potential (Tao et al., 2021; Whyte et al., 2012). Dysregulation of epigenetic pathways, including aberrant enhancer activity, is common in cancer (Helmsauer et al., 2020; Okabe and Kaneda, 2021).

Super enhancers (SEs) are large regions of chromatin that are densely bound by transcription factors and are strongly marked by H3K27ac (Parker et al., 2013; Whyte et al., 2013). Genes controlled by SEs are highly expressed and often sit at the apex of networks that establish cell identity (Whyte et al., 2013). Cancer cells, including neuroblastoma cells, are particularly sensitive to altered transcription of SE-controlled genes (Lovén et al., 2013). Identification of SEs is thus valuable for determining target points to disrupt tumors and understanding how SEs are dysregulated may provide insights regarding mechanisms of tumor initiation and progression.

There are two predominant biological states of neuroblastoma cells: an undifferentiated mesenchymal state and an adrenergic state more closely resembling differentiated, committed sympathetic neurons (Gartlgruber et al., 2021; van Groningen et al., 2017). Gene signatures associated with these states have prognostic value, with the mesenchymal phenotype being associated with worse outcomes, and relapsed lesions also being enriched for markers of the mesenchymal state (Gartlgruber et al., 2021; van Groningen et al., 2017). Tumor cell lines treated with radiation or chemotherapeutic agents have been shown to adopt a mesenchymal gene expression profile, a process which involves activation of NOTCH signaling (Boeva et al., 2017; van Groningen et al., 2019). This seemingly conflicts with longstanding evidence that post-treatment tumors often undergo histologic maturation (Finklestein et al., 1979; Grosfeld et al., 1978). However, despite histologic maturation with therapy, many patients relapse and succumb to their disease, raising the possibility that mature cells seen soon after therapy are replaced by cells with less differentiated features. Moreover, it is unclear whether neuroblastoma plasticity represents clonal evolution with shifting balances among distinct tumor subpopulations or plasticity of individual cells.

The present study was undertaken to determine whether neuroblastoma cells lacking *MYCN* amplification include heterogeneous cellular phenotypes. We investigated whether the cell-surface marker CD49b (integrin α2, encoded by *Itga2* in mice), which marks proliferative neural crest progenitor cells (Abe et al., 2016; Joseph et al., 2011), distinguishes neuroblastoma populations with distinct gene expression programs. We found that cells lacking CD49b expression were non-cycling, expressed RNAs encoding adrenergic transcription factors and transcribed neuronal marker genes. In contrast, CD49b-expressing cells were proliferative, transcribed transcription factor genes associated with the mesenchymal cell state and did not express neuronal genes. As expected, different complements of active enhancers and SEs were associated with these populations. Intriguingly, we found that cells with low expression of CD49b (CD49b-low) cells, which otherwise show many hallmarks of mature neurons, maintained the priming H3K4me1 mark at many enhancers and SEs that were active only in cells with high expression of CD49b (CD49b-high cells), suggesting that mature cells retain an abnormal ability to de-differentiate. Importantly, cells with either phenotype could give rise to the opposite cell type in culture. These results suggest that a bidirectional differentiation hierarchy exists in neuroblastoma, likely due to failure to decommission enhancer and SE elements. Defining the epigenetic mechanisms that restrict neural crest cell fate may thus be critical for understanding and treating this aggressive childhood disease.

## RESULTS

### Neuroblastoma cells express the integrin CD49b

**Fig. 1.**
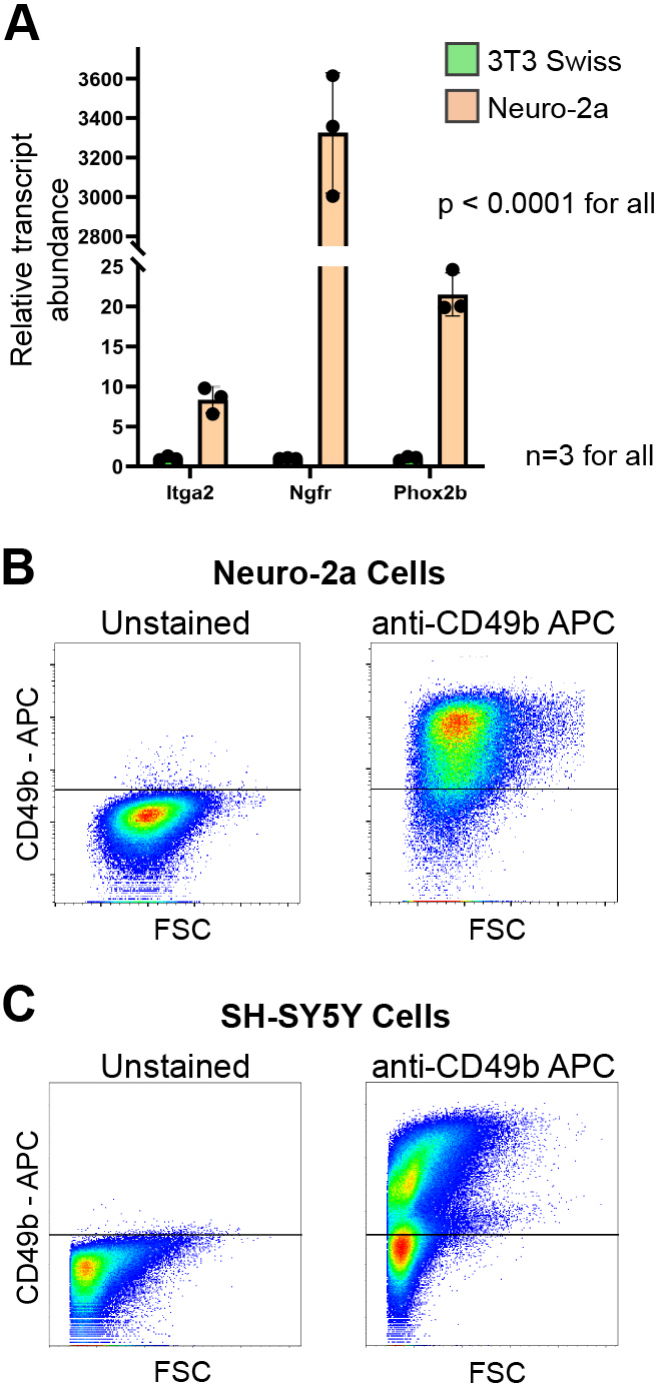
**Neuroblastoma cells express the CD49b cell-surface antigen.** (A) qPCR for the indicated genes in the mouse Neuro-2a (N2a) neuroblastoma cell line, as well as the mouse 3T3 Swiss fibroblast cell line. Bars indicate the mean, dots indicate individual replicates, and error bars indicate standard deviation. *Gapdh* expression was used for normalization. Two-tailed unpaired *t*-test was used to calculate *P*-values. (B,C) Representative flow cytometry plots showing CD49b antigen expression on mouse Neuro-2a (B) and human SH-SY5H (C) neuroblastoma cells. APC, allophycocyanin channel; FSC, forward scatter.

Because CD49b marks neuronal progenitor cells in neural crest-derived lineages but is not expressed on differentiated neurons (Belkind-Gerson et al., 2015; Joseph et al., 2011; Morarach et al., 2021), we hypothesized that this antigen would distinguish immature and mature neuroblastoma cells. We first assessed expression of the *Itga2* gene, which encodes the CD49b antigen, in mouse 3T3 Swiss fibroblast cells, as well as in the murine neuroblastoma cell line Neuro-2a (N2a), which lacks *Mycn* amplification. As a positive control, we also assessed the expression of *Ngfr* and *Phox2b*, two genes known to be highly expressed in neuroblastoma (Baker et al., 1989; Boeva et al., 2017). Consistent with our hypothesis, N2a cells displayed markedly higher transcript levels of *Itga2* than did 3T3 cells (Fig. 1A). We followed this result by assessing cell surface expression of CD49b on N2a cells, as well as on human SH-SY5Y cells, a patient-derived neuroblastoma cell line that also lacks *MYCN* amplification. Both lines were heterogeneous with respect to CD49b cell-surface expression (Fig. 1B,C). We noted that N2a cells had a continuum of expression, from cells lacking the antigen to those with high expression, whereas SH-SY5Y cells had more distinct CD49b-negative and CD49b-positive populations. This different staining pattern might reflect different expression patterns in human and mouse cells, or it might be an artifact of the different antibodies used.

### CD49b expression distinguishes neuroblastoma cell states

To determine whether variation in CD49b expression reflects the differentiation state of neuroblastoma cells, we sorted both N2a and SH-SY5Y cells based on CD49b expression. Because CD49b does not demarcate discrete populations of N2a cells, we sorted these cells into the lowest and highest expression quartiles based on staining for the antigen (Fig. S1A). We henceforth refer to these populations CD49b-negative (CD49b-neg) and CD49-high, respectively, as the lowest 25% of N2a cells based on CD49b staining displayed similar signal as that of the unstained control (Fig. S1A). The intermediate 50% of N2a cells, which expressed low levels of the antigen, are referred to as CD49b-low cells. In contrast, SH-SY5Y cells were sorted into distinct populations with and without detectable CD49b antigen, which we respectively refer to as Cd49b-positive (CD49b-pos) and CD49b-neg cells (Fig. S1B).

**Fig. 2.**
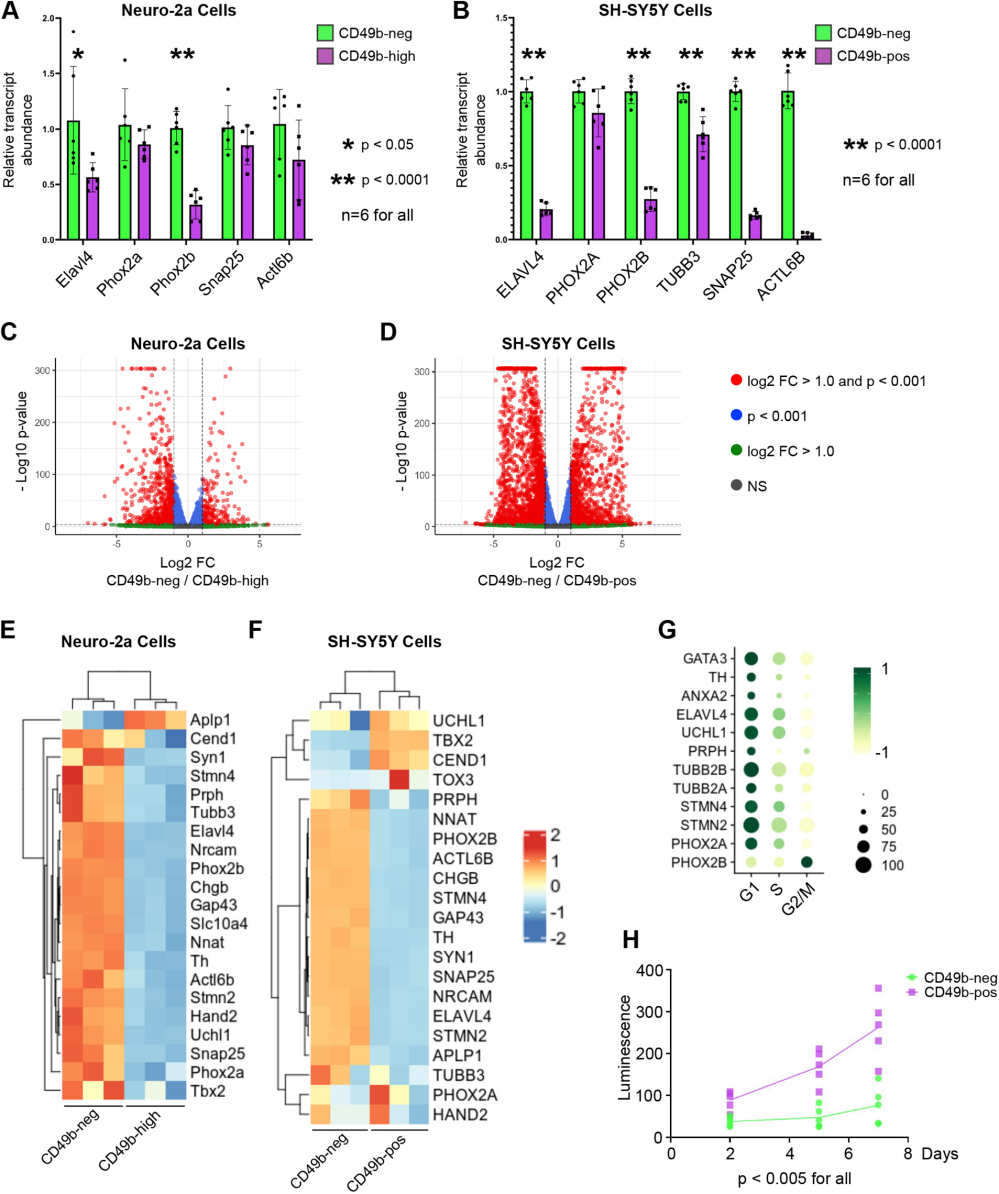
**CD49b expression distinguishes transcriptionally disparate subpopulations within neuroblastoma cell lines.** (A) qPCR for the indicated genes in N2a cells sorted into CD49b-neg and CD49b-high fractions. *Gapdh* expression was used for normalization. (B) qPCR for the indicated genes in SH-SY5Y cells sorted into CD49b-neg and CD49b-pos fractions. *GAPDH* expression was used for normalization. For A,B, bars indicate the mean, dots indicate individual replicates, and error bars indicate standard deviation. Two-tailed unpaired *t*-tests were used to calculate *P*-value. (C) Volcano plot showing differentially expressed genes in CD49b-neg N2a cells relative to CD49b-high N2a cells identified by poly(A)-enriched RNA sequencing. 4251 genes had |log_2_(fold change or FC)|>1 and *P*-value <0.001. (D) Volcano plot showing differentially expressed genes in CD49b-neg SH-SY5Y cells relative to CD49b-pos SH-SY5Y cells identified by poly(A)-enriched RNA sequencing. 8409 genes had |log_2_(fold change or FC)|>1 and *P*-value <0.001. NS, not significant. (E) Heatmap showing enrichment of selected neuronal marker genes in CD49b-high N2a cells relative to CD49b-neg N2a cells. (F) Heatmap showing enrichment of selected neuronal marker genes in CD49b-pos SH-SY5Y cells relative to CD49b-neg SH-SY5Y cells. For E,F, heatmaps reflect z-scores of log_2_-scale differences in gene expression. (G) Dot plot showing the proportion of cells expressing the indicated neuronal marker genes, stratified by cell cycle status inferred from gene expression patterns (data from GSE137804; Dong et al., 2020). Dot size indicates the proportion of cells expressing each gene, and color indicates the relative level of expression. (H) Proliferation in culture of SH-SY5Y cells sorted into CD49b-pos and CD49b-neg fractions (*n*=5). Two-tailed unpaired *t*-tests were used to calculate *P*-values.

After sorting, we isolated RNA and performed quantitative PCR (qPCR) for selected neuronal marker genes (Fig. 2A,B). Consistent with the role of CD49b as a marker of immature neuronal precursors in other neural crest-derived lineages, we found diminished expression of the neuronal genes *Elavl4*, *Phox2a*, *Phox2b*, *Snap25* and *Actl6b*, although only *Elavl4* and *Phox2b* reached statistical significance (*P*<0.05) in N2a cells (Fig. 2A). In SH-SY5Y cells, transcript levels of the human genes *ELAVL4*, *PHOX2A*, *PHOX2B*, *TUBB3*, *SNAP25* and *ACTL6B* were diminished, with all except *PHOX2A* reaching statistical significance (Fig. 2B).

We next performed poly(A)-enriched RNA sequencing (RNA-seq) on sorted CD49b populations in both cell lines. These experiments were undertaken to test, in an unbiased manner, whether CD49b marks biologically distinct neuroblastoma cells. We found striking differences, with 4251 genes in N2a cells and 8409 genes in SH-SY5Y cells achieving the predetermined thresholds of greater than 2-fold difference in gene expression and *P*<0.0001 (Fig. 2C,D). To query whether this reflects neuronal differentiation status, we compared our RNA-sequencing replicates based on the expression of selected neuronal marker genes. Consistent with our qPCR data, CD49b-neg cells in both the N2a and SH-SY5Y lines were markedly enriched for neuronal genes (Fig. 2E,F). We also examined genes encoding transcription factors that have been associated with the adrenergic and mesenchymal cell states in the literature (van Groningen et al., 2017). Genes encoding adrenergic factors, which include many genes associated with neurogenesis in the neural crest, were enriched in the CD49b-neg population in both cell lines (Fig. S2A). In contrast, genes encoding mesenchymal factors displayed notably higher transcript levels in the CD49b-high population in N2a cells and CD49b-pos population in SH-SY5Y cells (Fig. S2B). These data confirm that CD49b distinguishes distinct populations among neuroblastoma cells and that lack of CD49b indicates cells with a transcriptional program characteristic of differentiated neurons.

We then queried whether the distinct gene expression patterns seen in CD49b-neg and CD49b-pos cells might correlate with proliferation. Using a published single-cell RNA-seq (scRNA-seq) dataset of tumor cells from high-risk lesions lacking *MYCN* amplification (Dong et al., 2020), we inferred cell cycle status based on expression of cell cycle-associated genes and then assessed expression of numerous neuronal markers. Strikingly, except for *PHOX2B*, all the neuronal marker genes we assessed are enriched in non-cycling cells, with diminished transcript levels among cells in the S, G2 and M phases of the cell cycle (Fig. 2G). This strongly suggests that less mature neuroblastoma cells, which correspond to the CD49b-high/pos population in our cell lines, are proliferative, whereas cells expressing genes associated with mature neurons are non-cycling. We confirmed this result by sorting the human SH-SY5Y cell line into CD49b-pos and CD49b-neg populations, plating equal numbers of cells from each group, and assessing proliferation via a luminescence assay 2, 5 and 7 days later. The CD49b-pos population showed significantly greater proliferation at all time points (*P*<0.005; Fig. 2H).

### Distinct signaling pathways characterize cells distinguished by CD49b expression

**Fig. 3.**
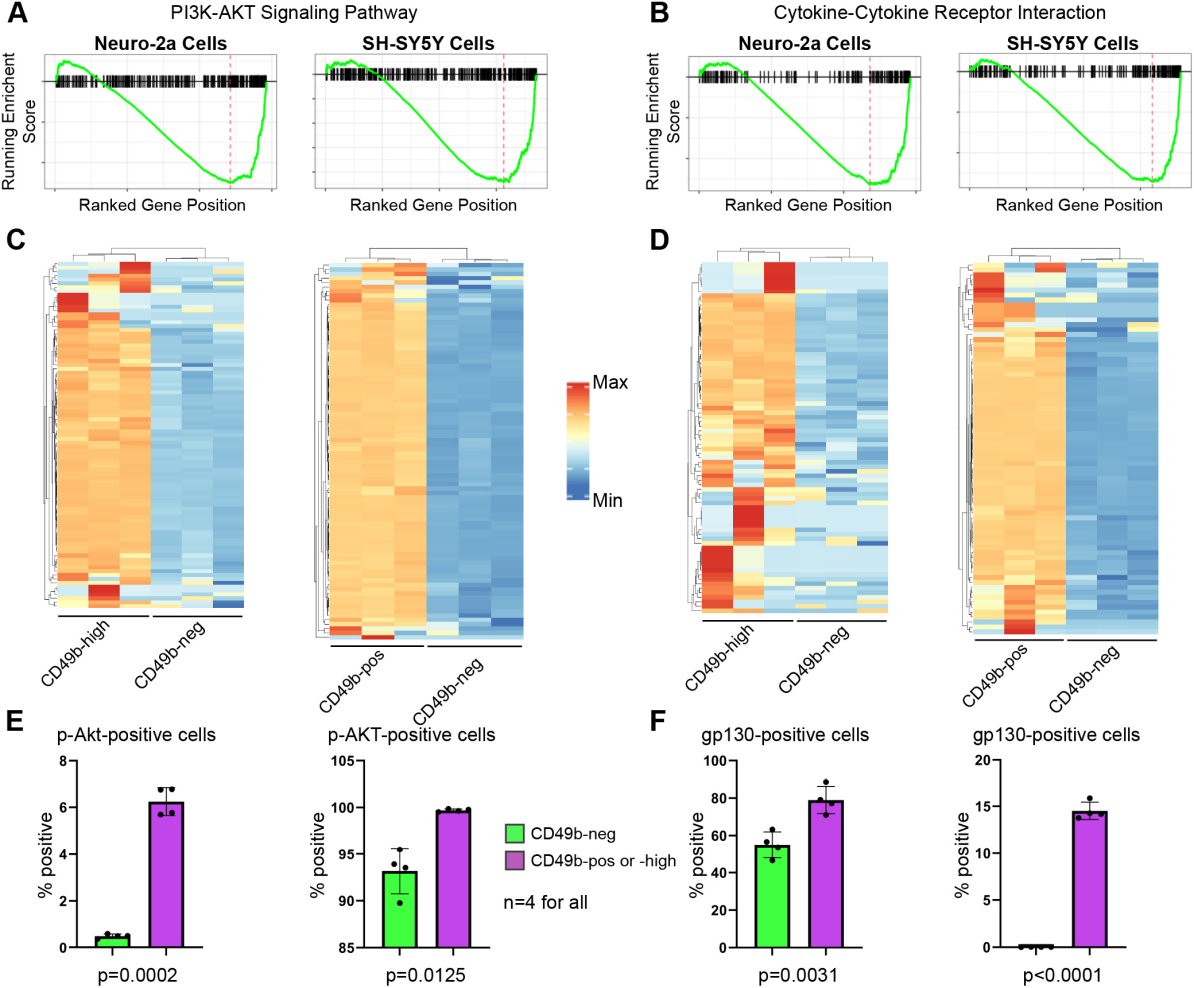
**CD49b differentiates neuroblastoma cells with different signaling milieus.** (A,B) GSEA enrichment plots showing enrichment of genes associated with the GSEA terms ‘PI3K-AKT signaling pathway’ (A) or ‘cytokine-cytokine receptor interaction’ (B) in CD49b-high N2a (left) or CD49b-pos SH-SY5Y (right) cells relative to CD49b-neg cells. The green lines represent the running enrichment score and the red dashed lines denote the point of maximum enrichment within the ranked gene list. (C,D) Heatmaps showing enrichment of genes associated the GSEA terms ‘PI3K-AKT signaling pathway’ (C) or ‘cytokine-cytokine receptor interaction’ (D) in CD49b-high N2a (left) or CD49b-pos SH-SY5Y (right) cells relative to CD49b-neg cells. (E,F) Quantification of flow cytometry analysis demonstrating increased p-Akt/p-AKT levels (E) and increased gp130 levels (F) in CD49b-expressing N2a (left) or SH-SY5Y (right) cells relative to CD49b-neg cells. Bars indicate mean, dots indicate individual replicates, and error bars indicate standard deviation. Two-tailed unpaired *t*-tests were used to calculate *P*-values.

To further validate that CD49b expression identifies distinct cell groups, we performed gene set enrichment analysis (GSEA) to identify biological pathways that are preferentially active in one population or the other. Using an adjusted *P*-value cutoff of 0.05, we identified 46 KEGG pathways that with differential enrichment between CD49b-neg and CD49b-high N2a cells, and 83 KEGG pathways with differential enrichment between CD49b-neg and CD49b-pos SH-SY5Y cells (Fig. S3, Tables S2 and S3). We noted that many of the pathways identified were conserved between these mouse and human cell lines. We focused on the PI3K-Akt and cytokine-cytokine receptor interaction pathways, both of which had significant differences in both cell lines and which are known to impact neuroblastoma cell phenotypes (Cotterman and Knoepfler, 2009; Hatzi et al., 2002; Paul et al., 2013). Enrichment plots and heatmaps of genes associated with these pathways showed significant enrichment in the CD49b-high and CD49b-pos populations in N2a and SH-SY5Y cells, respectively (Fig. 3A-D). Using flow cytometry, we confirmed that in both cell lines, CD49b-high/pos cells had higher levels of active, phosphorylated (p-) Akt/AKT, and a higher percentage of cells expressed the gp130 (IL6ST) cytokine receptor (Fig. 3E,F). These results provide additional evidence that CD49b expression distinguishes biologically distinct neuroblastoma cells.

### CD49b-neg N2a cells maintain H3K4me1 priming marks at enhancers active in CD49b-high cells

**Fig. 4.**
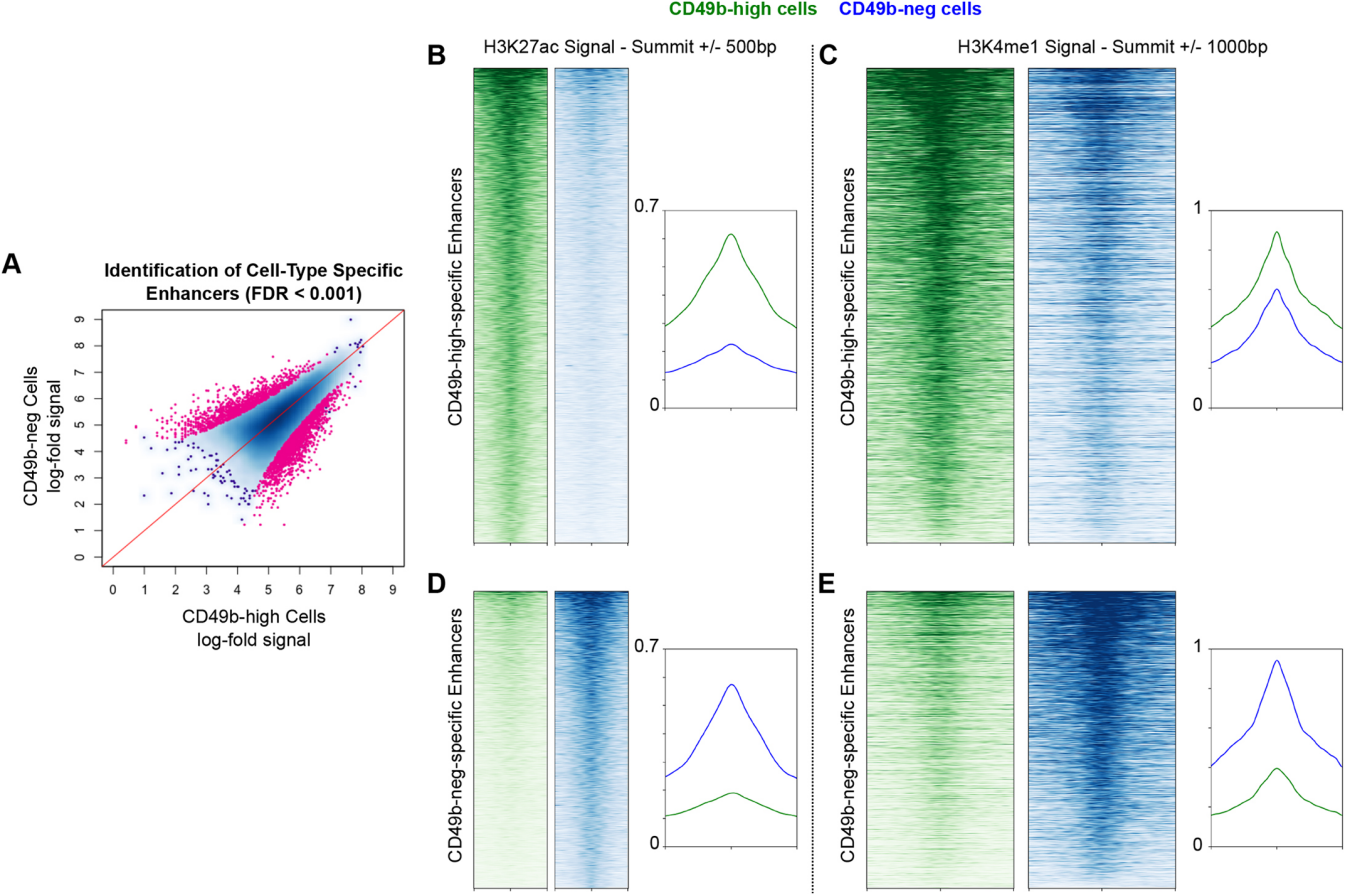
**CD49b-neg cells have a distinct repertoire of active enhancers but maintain the priming H3K4me1 mark on enhancers active in CD49b-high cells.** (A) Identification of enhancers with greater H3K27ac signal in CD49b-neg or CD49-high N2a cells. Greater distance below the red line indicates greater specificity for CD49b-high cells, and greater distance above the red line indicates greater specificity for CD49b-neg cells. Enhancers with a false discovery rate <0.001 were deemed specific to one population. (B-E) Heatmap and profile plot showing H3K27ac (B,D) or H3K4me1 (C,E) signals at CD49b-high-specific enhancers (B,C) or CD49b-neg-specific enhancers (D,E) in CD49b-high (green) and CD49b-neg (blue) cells. Heatmaps are centered at enhancer summits and show 500 base pairs (B,D) or 1000 base pairs (C,E) upstream and downstream.

Gene expression is determined by the enhancer elements active in cells. Given the striking differences in gene expression among subpopulations of neuroblastoma cells, we hypothesized that cells with different levels of CD49b antigen expression would have distinct enhancer profiles. Because acquisition of a neuronal gene signature, including *ACTL6B* (*Actl6b* in mice), is associated with terminal differentiation during normal neurogenesis (Morarach et al., 2021; Yoo et al., 2017), we also hypothesized that CD49b-neg cells would decommission enhancers that are active in CD49b-pos/high cells. We used CUT&RUN to globally assay H3K27ac and H3K4me1 in CD49b-neg and CD49b-high populations of N2a cells. We first identified enhancers active in each population by assessing H3K27ac signals. Using a false discovery rate (FDR) of <0.001, we identified 3622 enhancers specifically active in one population or the other, including 2225 enhancers active in CD49b-high cells and 1397 enhancers active in CD49b-neg cells (Fig. 4A). As anticipated, enhancer regions activated in each population displayed markedly diminished H3K27ac signals in the other population (Fig. 4B,D). However, when we examined the signals of the priming mark H3K4me1 at the same loci, we found that CD49-neg cells maintained this mark, albeit to a slightly diminished extent, at enhancers active in CD49b-high cells (Fig. 4C). Contrary to our expectation, CD49b-neg cells thus maintained CD49b-high-specific enhancers in a poised state. A similar trend was observed at CD49b-neg enhancers, although to a lesser degree (Fig. 4E). This implies that CD49b-neg neuroblastoma cells, despite having a gene expression program characteristic of differentiated neurons, remain primed to de-differentiate to an immature state.

### CD49b-neg N2a cells maintain H3K4me1 markers at SEs that define the CD49b-high state

**Fig. 5.**
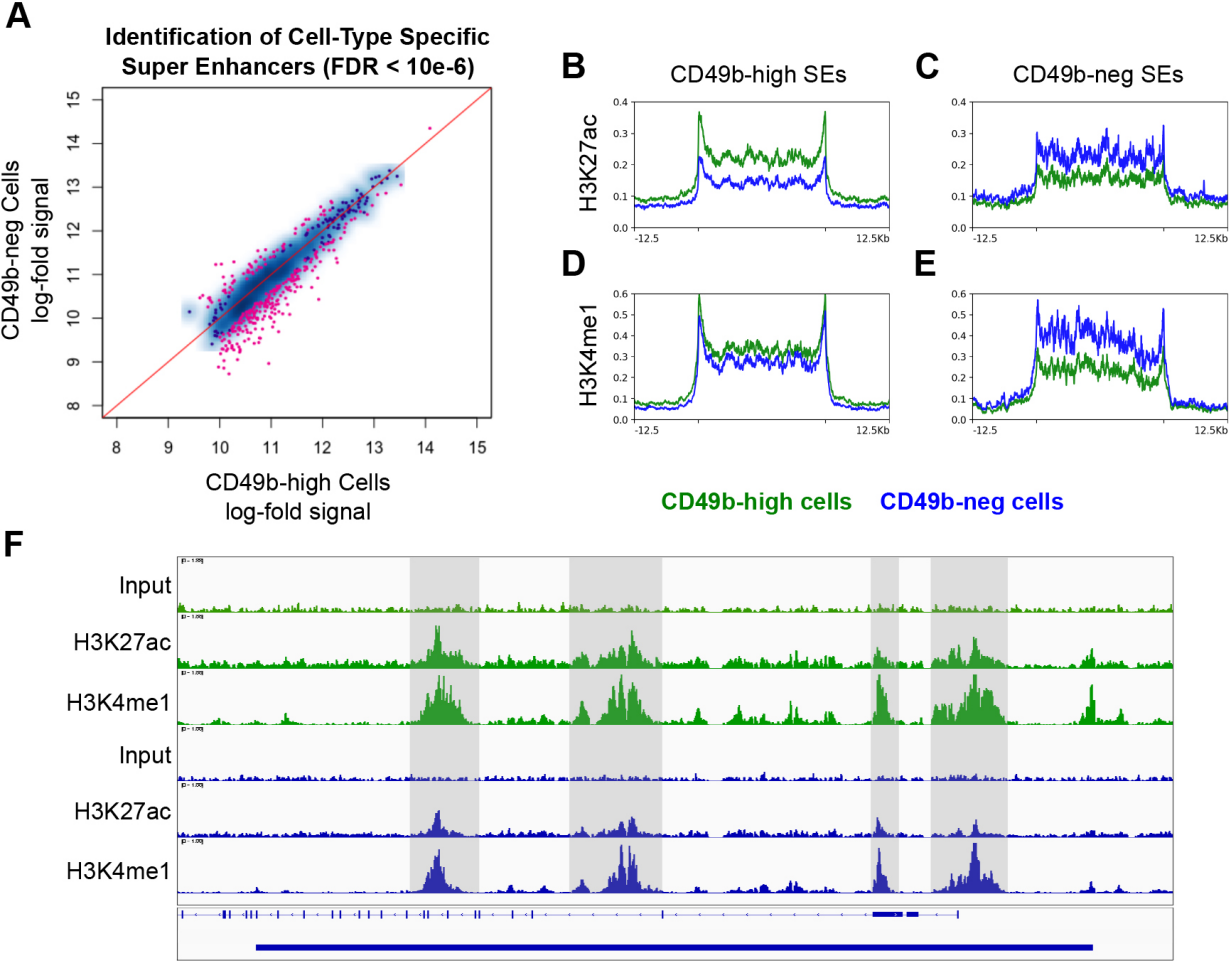
**CD49b-neg cells have a distinct super enhancer profile but maintain H3K4me1 marks on super enhancers active in CD49b-high cells.** (A) Identification of super enhancers (SEs) with greater H3K27ac signal in CD49b-neg or CD49-high cells. Greater distance below the red line indicates greater specificity for CD49b-high cells, and greater distance above the red line indicates greater specificity for CD49b-neg cells. SEs with a false discovery rate <0.000001 were deemed specific to one population. (B-E) Profile plots showing H3K27ac (B,C) and H3K4me1 (D,E) signals at CD49b-high-specific SEs (B,D) or CD49b-neg-specific SEs (C,E) in CD49b-high (green) and CD49b-neg (blue) cells. SEs are scaled to 25,000 base pairs, and 12,500 base pairs upstream and downstream are shown. (F) Tracks plot showing signals for H3K27ac, H3K4me1 and input at the *Itga2* SE in N2a cells. CD49b-high cells are green, CD49b-neg cells are blue. The solid blue bar at the bottom indicates the SE region, and the hashed blue line indicates the *Itga2* coding region. Shaded gray regions were hand-selected to highlight diminished H3K27ac signal in CD49b-neg cells despite maintenance of the H3K4me1 signal. All windows are scaled equally.

SEs are large regions of chromatin that often overlap genes controlling cell identity. We used the Rank Ordering of Super-Enhancers (ROSE) algorithm (Whyte et al., 2013) to identify all SEs active in N2a cells, and then used DESeq2 with a stringent FDR (<10^−6^) to identify SEs active in the CD49b-neg and CD49b-high populations. This approach identified 69 SEs that are active only in CD49b-neg cells and 228 SEs active only in CD49b-high cells (Fig. 5A). Profile plots revealed that H3K27ac signals were diminished in each population at SE loci that were active in the opposite cell type (Fig. 5B,C). This was also seen for H3K4me1 signals in SEs active in CD49b-neg cells (Fig. 5E). However, in CD49b-neg cells, H3K4me1 signals at CD49b-high SE loci were only slightly diminished (Fig. 5D). This is illustrated by the SE region overlapping the *Itga2* transcriptional start site, which is one of the SEs that defines the CD49b-high cell state (Fig. 5F). Taken together with the data in Fig. 4, these results indicate that CD49b-neg neuroblastoma cells are epigenetically poised to activate the enhancers and SEs that determine the CD49b-high state.

### Neuroblastoma cells can interconvert between CD49b expression states *in vitro*

**Fig. 6.**
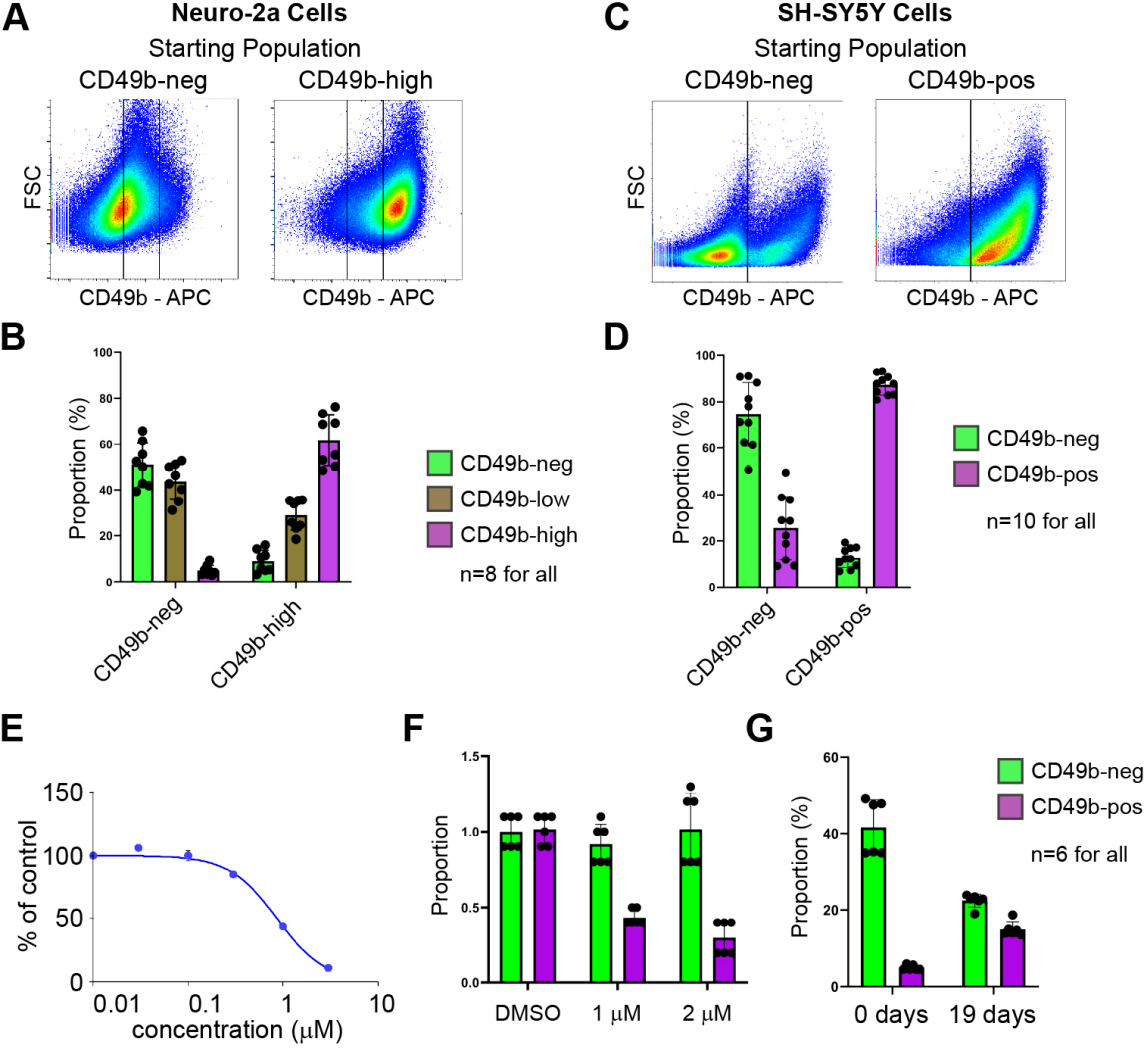
**Neuroblastoma cells transition between CD49b expression profiles in culture.** (A,C) Representative flow cytometry plots showing reanalysis of N2a cells (A) and SH-SY5Y cells (C) at 7 and 21 days, respectively, after cultures were initiated with the indicated starting populations. APC, allophycocyanin channel; FSC, forward scatter. (B,D) Quantification of the proportion of N2a cells (B) and SH-SY5Y cells (D) in each CD49b expression category at 7 and 21 days, respectively, after cultures were initiated with the indicated starting populations. (E) Dose response curve showing proportion of N2a cells surviving following 48 h of treatment with the indicated concentrations of doxorubicin, as measured by the CellTiter-Glo assay. (F) Proportion of total N2a cells within the CD49b-neg and CD49b-high gates after treatment with DMSO (vehicle) or 1 µM or 2 µM of doxorubicin for 48 h, as measured by flow cytometry. (G) Proportion of N2a cells that were CD49b-neg and CD49b-high at the conclusion of 48 h of treatment with 1 µM doxorubicin, and after 19 days of recovery in culture. Bars indicate the mean, dots indicate individual replicates, and error bars indicate standard deviation.

Given the persistence of H3K4me1 marks at CD49b-high-specific enhancers in CD49b-neg N2a cells, we hypothesized that these cells can switch to a CD49b-high state. To test this, we sorted N2a cells and SH-SY5Y cells using the gating strategies shown in Fig. S1, then returned the sorted populations to culture under normal growth conditions for 7 days (N2a cells) or 21 days (SH-SY5Y cells). Cells were then reanalyzed for CD49b expression by flow cytometry. Because N2a cells show a continuum of CD49b expression, we included cells that were neither CD49b-neg nor CD49b-high in our analysis and referred to this middle population as CD49b-low (Fig. S1A). Consistent with our hypothesis, cultures beginning with a pure population of CD49b-neg cells generated large numbers of cells expressing the antigen in both N2a cells (Fig. 6A,B) and SH-SY5Y cells (Fig. 6C,D). Interestingly, enhancer priming via H3K4me1 may not be necessary for plasticity, as cultures of CD49b-high N2a cells, which do not have strong H3K4me1 signals at CD49b-neg enhancers (Fig. 4E), also gave rise to cells lacking the antigen (Fig. 6A-D), suggesting that the CD49b-high to CD49b-neg switch involves *de novo* enhancer selection.

We sought to determine whether this ability to interconvert between CD49b expression states might account for relapse following cytotoxic therapy. We tested toxicity of several doses of the chemotherapy drug doxorubicin on N2a cells in culture and found an IC_50_ of 0.9 µM after 48 h of treatment (Fig. 6E). Treatment with either 1 µM or 2 µM of doxorubicin selectively eliminated CD49b-high cells from N2a cultures, consistent with the action of the cytotoxic agent against dividing cells (Fig. 6F). Immediately after withdrawal of doxorubicin, cultures of N2a cells contained very few CD49b-high cells, but after 19 days, recovery of this population was apparent (Fig. 6G). These data indicate that cell phenotype plasticity may be a mechanism for neuroblastoma cells to evade cytotoxic treatment.

### Retinoic acid treatment inhibits reversion to the immature, CD49b-high cell state

Retinoic acid (RA) has been used, with some success, as a clinical adjunct for neuroblastoma therapy (Matthay et al., 1999) and can induce neuronal differentiation of neuroblastoma cells in culture (Påhlman et al., 1984). We thus queried whether RA treatment would inhibit reversion of CD49b-neg N2a cells to a CD49b-high state. We sorted for CD49b-neg cells and returned this sorted population to culture. Cells were supplemented with either 10 µM RA or vehicle control (DMSO). After 1 week, we repeated flow cytometry analysis to assess recovery of the CD49b-high population. This experiment was performed twice, with different batches of N2a cells. RA treatment consistently inhibited reversion to the CD49b-high phenotype in both experiments (Fig. S4).

### CD49b-neg cells switch to a CD49b-high phenotype and generate tumors *in vivo*

**Fig. 7.**
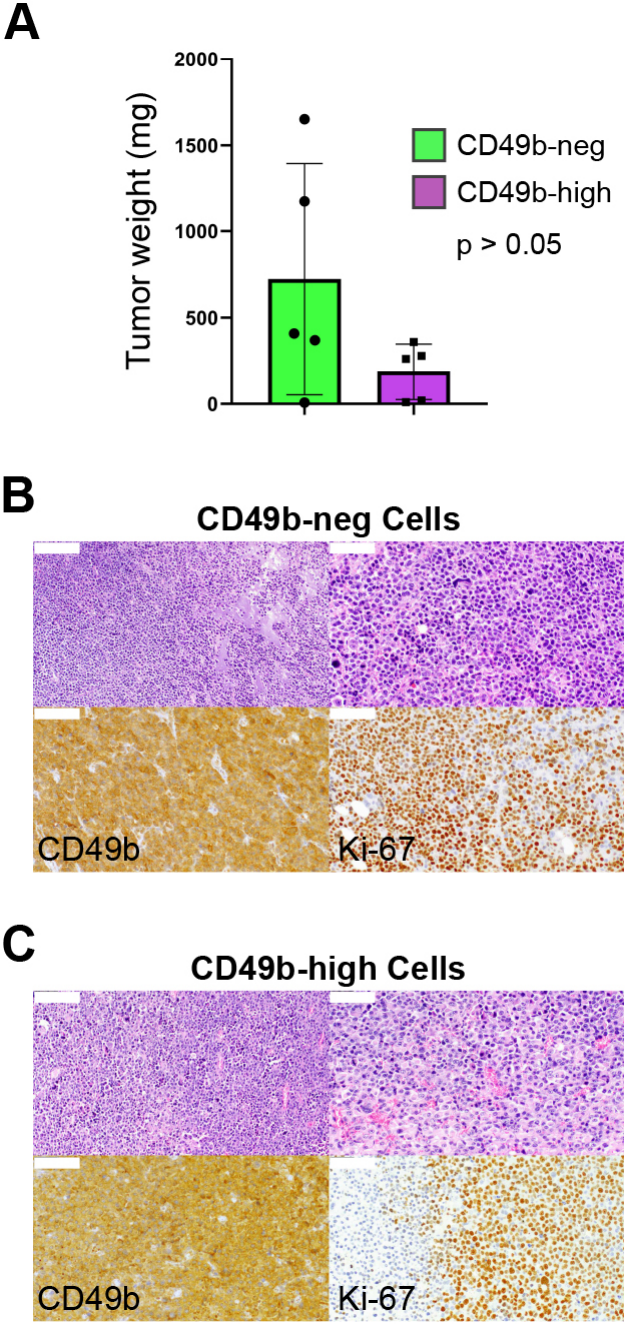
**CD49b-neg cells and CD49b-high cells both form CD49b-expressing tumors *in vivo*.** (A) Tumor weight immediately after euthanizing mice. Bars indicate the mean, dots indicate individual replicates, and error bars indicate standard deviation. Two-tailed unpaired statistical tests were used to calculate the *P*-values. (B,C) Microscopic examination of tumors grown from CD49b-neg (B) and CD49b-high (C) cells. High-power images (top right) of Hematoxylin and Eosin stains (top left) show that there was more eosinophilic cytoplasm in tumors grown from CD49b-high cells (C) than in tumors grown from CD49b-neg cells (B). Immunostaining for CD49b (bottom left) and Ki-67 (bottom right) shows that the tumors from Cd49b-neg cells (B) and from Cd49b-high cells (C) were diffusely reactive for CD49b and highly proliferative, except in areas of necrosis (C). Images are representative of tumors grown in five mice per group. Scale bars: 200 µm (top left) and 100 µm (top right, bottom left and bottom right).

We next sought to determine whether neuroblastoma cells exhibit similar phenotypic plasticity *in vivo*. The N2a cell line is derived from a spontaneous tumor in A/J mice and, when injected into syngenic animals, forms rapidly growing tumors in a native microenvironment (Lee et al., 2012; Srinivasan et al., 2018). Taking advantage of this model, we sorted N2a cells into CD49b-neg and CD49b-high populations and then injected 2×10^5^ cells per animal into the flank of A/J mice. Mice were euthanized 10 days later. Surprisingly, there was no statistically significant difference in weight between tumors grown from CD49b-neg and CD49b-pos cells, although there was greater variability in tumor size in the CD49b-neg group (Fig. 7A). Histological examination of the tumors revealed a lack of neuropil in all cases, suggesting poorly differentiated neuroblastomas, although an eosinophilic cytoplasm did appear more abundant in CD49b-high tumors (Fig. 7B,C). Interestingly, both sets of tumors were diffusely positive for CD49b (Fig. 7B,C), confirming that CD49b-neg cells can generate tumors with a CD49b-high phenotype *in vivo*. Both sets of tumors were also diffusely positive for Ki-67 (Fig. 7B,C), indicating significant cell proliferation, although Ki-67 staining was diminished in areas of necrosis (Fig. 7C).

## DISCUSSION

Recent studies have shown that neuroblastoma cells have two predominant states, a differentiated adrenergic state characterized by neuronal transcripts and a less-differentiated mesenchymal state expressing genes seen in neural crest cells. Prior studies assessing these states in neuroblastoma cell lines have sequenced bulk, unsorted populations, making it impossible to investigate heterogeneity within populations (Gartlgruber et al., 2021; van Groningen et al., 2017). Our work establishes that two neuroblastoma cell lines lacking *MYCN* amplification, murine N2a and human SH-SY5Y, contain cells with gene expression patterns characteristic of both the adrenergic and mesenchymal states. We have also shown that N2a and SH-SY5Y cells are plastic between these states. It is known that treating neuroblastoma cells with cytotoxic agents can alter gene expression (Boeva et al., 2017), but prior research in this area has relied on bulk expression profiles, so until now it has not been clear whether individual cells are plastic. We have now shown that sorted populations of neuroblastoma cells revert to a mixed population, meaning that cells either directly switch phenotypes or undergo asymmetric divisions producing disparate daughter cells. Reversion towards equilibrium proportions from sorted starting populations has been shown previously for cancer cells (Gupta et al., 2011), but to our knowledge this is the first demonstration in neuroblastoma.

We have shown that the adrenergic and mesenchymal states can be distinguished by expression of the CD49b antigen. CD49b is an integrin that marks immature neuronal precursors in the peripheral nervous system (Belkind-Gerson et al., 2015; Joseph et al., 2011). Downregulation of this antigen is associated with a transition to a committed neuronal state, but reversion of CD49b-negative neurons to a CD49b-expressing state has not been observed during normal development. The ability of tumor cells to revert from a CD49b-neg state, which is characterized by expression of neuronal genes, inverts the normal trajectory of neurogenesis. We found that CD49b-neg N2a cells maintained the priming H3K4me1 mark at enhancer loci that were active in CD49b-high cells, which may explain their ability to revert to a less mature gene expression state. Decommissioning of primed enhancers appears to be a key mechanism for cells to maintain differentiation (Tao et al., 2021; Whyte et al., 2012). We thus speculate that oncogenic mutations in neuroblastoma likely act, at least in part, by preventing proper decommissioning of enhancer elements. The recent report that loss of the tumor suppressor gene *ARID1A* causes de- differentiation of neuroblastoma cells supports this hypothesis (Shi et al., 2020).

SEs are large genomic regions marked heavily by H3K27ac and densely bound by transcription factors (Parker et al., 2013; Whyte et al., 2013). These regions control cell fate-determining genes, and disruptions of SEs occur in cancers including neuroblastoma (Gartlgruber et al., 2021; Lovén et al., 2013; van Groningen et al., 2017). We found that neuroblastoma cells marked by high or low CD49b expression had distinct SE profiles, which is strong evidence that the CD49b antigen distinguishes different biological states. Additionally, one of the SEs we identified in CD49b-high cells overlaps with the *Itga2* gene, which encodes the CD49b antigen. This suggests that CD49b is not merely a marker of proliferative, immature cells, but may also have an important role in establishing such a state. Intriguingly, CD49b activates the AKT pathway in esophageal squamous cell carcinoma and hepatocellular carcinoma (Huang et al., 2021; Juratli et al., 2022), suggesting a mechanistic link between the antigen and a correlated signaling network. Although functional studies of CD49b are outside the scope of this report, we anticipate that it will be an interesting avenue for future studies.

Although SEs active in CD49b-high N2a cells had diminished H3K27ac signals in CD49b-low N2a cells, we found these same SEs to maintain high levels of H3K4me1. Similarly, maintenance of the H3K4me2 priming mark at SEs has been observed across macrophage subtypes (Gosselin et al., 2014). H3K4me1 marks act by recruiting the BAF complex to ordinary enhancers (Local et al., 2018), where the BAF complex establishes accessible chromatin as a key step in enhancer activation (Vierbuchen et al., 2017). To our knowledge, whether the H3K4me1 mark plays a priming role in SEs as well as at traditional enhancers has not been explored. We speculate that multipotent cells may maintain alternate fate potentials by monomethylating or dimethylating H3K4 in SE regions, thus recruiting the BAF complex and generating open chromatin that can be rapidly activated to initiate expression of fate-determining transcripts. This model would correlate with the recent description of domains of regulatory chromatin (DORCs), which are large regions around fate-determining genes with an open chromatin structure (Ma et al., 2020). DORCs correlate highly with SEs identified by H3K27ac signals, and chromatin accessibility at DORCs generally precedes gene expression. Future work to test these hypotheses and establish the underlying mechanisms could yield valuable insights for neuroblastoma therapy.

Intriguingly, RA has been used clinically as an adjunct to cytotoxic therapy, with some data suggesting improved outcomes (Matthay et al., 1999). When added to the cell culture medium, RA can induce neuronal differentiation of neuroblastoma cell lines (Påhlman et al., 1984) and recent work suggests that this involves epigenetic activation of neuronal fate-determining genes (Zimmerman et al., 2021). In the present work, we show that RA treatment significantly inhibits reversion of CD49b-neg N2a cells to an immature, CD49b-high state. We speculate that this RA- mediated effect occurs through alteration of the epigenome of neuroblastoma cells. Further studies are needed to determine the mechanisms through which RA impacts neuroblastoma tumor cell differentiation.

This study has several limitations. Our results are derived from two tumor cell lines, one mouse and one human, that lack *MYCN* amplification. Further work utilizing primary tumor specimens will be important as cell lines maintained in long-term culture do not always faithfully model human disease. The shortcomings of using cell lines for this study are partly alleviated by correlating our results with published scRNA-seq data. Some genes that were differentially expressed in our RNA-seq data did not achieve statistical significance at the predetermined *P*-value cutoff of 0.05 in qPCR experiments, although all genes analyzed did show a trend in expression consistent with the RNA-seq results. Additionally, as the biology of *MYCN*-amplified and non-*MYCN*-amplified tumors differ, our results may be irrelevant to the large subset of patients with *MYCN* amplification. Because we used a bulk sequencing approach to profile histone modifications in CD49b-neg and CD49b-high N2a populations, unappreciated heterogeneity within these populations may exist. Finally, although we demonstrated switching of CD49b-negative N2a cells to the CD49b-high phenotype with a syngenic animal model, changing in the opposite direction was not observed *in vivo*. Additional work to understand behavior of neuroblastoma tumor cells in the complex tumor environment of patients and animal models will be critical.

## MATERIALS AND METHODS

### Cell lines and cell culture

Mouse 3T3 Swiss (CCL-92), mouse N2a (CCL-131) and human SH-SY5Y (CRL-2266) cells without mycoplasma detection were obtained from American Type Culture Collection (ATCC, Manassas, VA, USA). SH-SY5Y cell identity was confirmed by short tandem repeat testing by ATCC. 3T3 cells were maintained in Dulbecco's modified Eagle medium (DMEM, 11965-118 Thermo Fisher Scientific, Waltham, MA, USA) supplemented with 10% fetal bovine serum (FBS, 25-550, Genesee Scientific, El Cajon, CA, USA) and 1% penicillin-streptomycin. N2a cells were maintained in Eagle's minimum essential medium (EMEM, 12-611F, Lonza, Basil, Switzerland) supplemented with 10% FBS and 1% penicillin-streptomycin. SH-SY5Y cells were maintained in DMEM/F12 (1:1) (11330-032, Thermo Fisher Scientific) supplemented with 15% FBS and 1% penicillin-streptomycin. Cells were monitored daily and passaged when they reached 80% confluency. All experiments were performed on cells less than ten passages from delivery from ATCC.

### Flow cytometry and cell sorting

All flow cytometry and cell sorting was performed at the Harvard Stem Cell Institute Center for Regenerative Medicine Flow Cytometry Core facility on FACSAria instruments (BD Biosciences, East Rutherford, NJ, USA). Cells were trypsinized, spun down, resuspended in PBS containing 10% FBS, and counted. Cells were blocked in PBS with 10% FBS on ice for 20 mins, and stained for 20 mins on ice with the following antibodies: anti-mouse CD49b (1:500, 103511, BioLegend, San Diego, CA, USA), anti-human CD49b (1:750, 359310, BioLegend), anti-mouse gp130 (1:100, 149404, BioLegend), anti-human gp130 (1:100, 362010, BioLegend) and anti-phospho-Akt (1:50, 4071, Cell Signaling Technology, Danvers, MA, USA). Scatter profiling and DAPI staining were used to exclude debris, doublets and dead cells. Gating for analysis was performed as shown in Fig. S1. All analysis was conducted using FlowJo software (BD Biosciences).

### qPCR

Total RNA was isolated from cells using RNEasy mini kits (Qiagen, Germantown, MD, USA). Reverse transcription and cDNA amplification were done using the iTaq Universal SYBR Green One-Step Kit (Bio-Rad, Hercules, CA, USA) and a CFX96 real-time system (Bio-Rad). Primer sequences for the murine genes *Itga2*, *Ngfr*, *Elavl4*, *Phox2a*, *Phox2b*, *Snap25*, *Actl6b* and *Gapdh* (normalization control) and for the human genes *ELAVL4*, *PHOX2A*, *PHOX2B*, *SNAP25*, *ACTL6B*, *TUBB3* and *GAPDH* (normalization control) were obtained from the Harvard Medical School PCR PrimerBank. Primer sequences are included in Table S1.

### RNA-seq and data analysis

Cells were isolated by sorting, after which total RNA was isolated using RNEasy mini kits (Qiagen) and treated with RNase-free DNase (Qiagen). RNA was quantified using the Qubit RNA HS Assay Kit (Thermo Fisher Scientific). Poly(A)-enriched libraries for sequencing were then generated using the NEBNext Ultra II Directional RNA Library Prep Kit for Illumina and the NEBNext Poly(A) mRNA Magnetic Isolation Module (New England Biolabs, Ipswich, MA, USA). Paired-end sequencing was performed on the Illumina NextSeq instrument at the Harvard University Bauer Core. Reads were aligned to the mm10 (mouse) and hg38 (human) reference genomes using STAR 2.7.3 on the Mass General Brigham ERISOne Cluster. Counts were computed using featureCounts 2.0.3 (https://github.com/ziquanzhao/FeatureCounts-2.0.3) on the ERISOne Cluster. Differential gene expression analysis was performed using the DESeq2 package (https://bioconductor.org/packages/release/bioc/html/DESeq2.html) in R 4.1.0, with count normalization done by the default median of ratios method in DESeq2. Heatmaps were generated with the ComplexHeatmap R package (https://bioconductor.org/packages/release/bioc/html/ComplexHeatmap.html), and volcano plots were generated with the EnhancedVolcano R package (https://bioconductor.org/packages/release/bioc/html/EnhancedVolcano.html). GSEA was performed using the clusterProfiler R package (https://bioconductor.org/packages/release/bioc/html/clusterProfiler.html).

### CUT&RUN and data analysis

Cells were isolated by sorting, after which the CUT&RUN assay was performed using the CUT&RUN Assay Kit (Cell Signaling Technology). The following antibodies were used at 1:50 dilution: anti-H3K4me1 (5326, Cell Signaling Technology) and anti-H3K27ac (8173, Cell Signaling Technology). Sequencing libraries were built using the DNA Library Prep Kit for Illumina (Cell Signaling Technology). Paired-end sequencing was performed on the Illumina NextSeq instrument at the Harvard University Bauer Core. Reads were aligned to the mm10 (mouse) and hg38 (human) reference genomes using Bowtie 2.4.1 (https://sourceforge.net/projects/bowtie-bio/files/bowtie2/2.4.1/) on the Mass General Brigham ERISOne Cluster. SAM file outputs by Bowtie were converted to the BAM format using Samtools View in the Samtools 1.4.1 package (https://sourceforge.net/projects/samtools/files/samtools/1.4.1/), and BAM files were filtered for uniquely mapped reads using Sambamba 0.4.7 (https://github.com/biod/sambamba/releases/tag/v0.4.7). BAM files were then randomly downsampled to contain equivalent numbers of reads using Samtools View. Peak calling was performed on downsampled BAM files using the MACS2 algorithm version 2.2.7.1 (https://pypi.org/project/MACS2/2.2.7.1/). H3K27ac peaks with differential signals in CD49b-neg and CD49b-high cells were then identified using the DiffBind package (https://bioconductor.org/packages/release/bioc/html/DiffBind.html) in R 4.1.0. SEs were identified using the ROSE algorithm on downsampled H3K27ac BAM files (Whyte et al., 2013) in Python 2.7.3, and SEs specifically active in CD49b-neg and CD49-high cells were then identified using DiffBind. Heatmaps and profile plots were generated using deepTools 3.5.1 (https://github.com/deeptools/deepTools/releases/tag/3.2.1). Genome browser tracks were created with the Integrative Genomics Viewer 2.11.1 (Robinson et al., 2011).

### Published scRNA-seq data

Previously published scRNA-seq data of primary neuroblastoma lesions were queried (Dong et al., 2020). Data are available from the NCBI Gene Expression Omnibus under the accession GSE137804. Raw data were obtained from the NCBI Sequence Run Archive using the SRA Toolkit ‘fastq-dump’ command. Genome alignment and feature-barcode matrix generation were performed with the Cell Ranger ‘cellranger count’ command on the Mass General Brigham ERISOne Cluster. Further analysis was performed with Seurat (https://satijalab.org/seurat/) in the R environment. Tumor cells from patients with high-risk disease and without *MYCN* amplification were identified based on cellular barcodes provided in the metadata from the depositing authors. Cells were processed and cell cycle status was annotated using the standard Seurat workflow. The dot plot was generated using the DotPlot function in the Seurat package.

### Cell proliferation assay

Approximately 5×10^3^ SH-SY5Y cells were plated in each well of 48-well culture dishes after sorting into CD49b-neg and CD49b-pos populations. At the indicated time points, cell viability was assayed using the CellTiter-Glo Luminescent Cell Viability Assay (Promega, Madison, WI, USA).

### Doxorubicin sensitivity assay

Approximately 5×10^3^ N2a cells were plated in each well of 96-well culture dishes. After 24 h, several doses of doxorubicin (PHR1789-200MG, Millipore Sigma, Burlington, MA, USA) were applied to the cells for 48 h. Cell viability was assayed using the CellTiter-Glo Luminescent Cell Viability Assay.

### RA treatment assay

After sorting cells as described above, 5×10^5^ CD49b-neg N2a cells were plated in two T25 cell culture flasks. The growth mediam was supplemented with either 10 μM all-trans RA (Sigma-Aldrich, Burlington, MA, USA) or vehicle (DMSO). Cells were passaged after 3 days, and the medium was refreshed again 3 days after passaging. Seven days after sorting, cells were reanalyzed by flow cytometry to assess the proportion of CD49b-high cells in each culture.

### *In vivo* tumor model

All animal procedures were approved by the Institutional Animal Care and Use Committee at Massachusetts General Hospital. A/J albino mice (strain 000646, Jackson Laboratory, Bar Harbor, ME, USA) at 3 weeks of age were injected on the right flank with 10^5^ N2a cells sorted into either CD49b-neg or CD49b-high populations. Five mice were injected with each group of cells. Ten days after injection, mice were euthanized and tumors were explanted. Tumors were weighed and fixed overnight in 10% formalin, after which they were transferred to 70% ethanol for long-term storage.

### Tumor histology

Fixed tumors were sectioned, mounted on slides, and stained with Hematoxylin and Eosin or with antibodies against Ki-67 (1:100, 652402, BioLegend) or CD49b (1:100, PA5-96739, Invitrogen) by the staff at the Histopathology Research Core at Massachusetts General Hospital. Slides were reviewed and imaged by a board-certified pathologist with expertise in neuroblastoma (K.M.C.).

### Statistical analysis

Statistical comparisons were performed using the unpaired, two-tailed *t*-test with *P*<0.05 set as the predetermined level of significance. Statistical testing was performed using GraphPad Prism (San Diego, CA, USA).

## Supplementary Material

10.1242/dmm.049754_sup1 Supplementary information
