# Supplementary material for: Differentiated neuroblastoma cells remain epigenetically poised for de-differentiation to an immature state
Source: Dis Model Mech. 2023 Dec 27;16(12):dmm049754. doi: 10.1242/dmm.049754 (PMC10810560; doi:10.1242/dmm.049754)
Supplement: Supplementary information [file dmm-16-049754-s1.pdf]

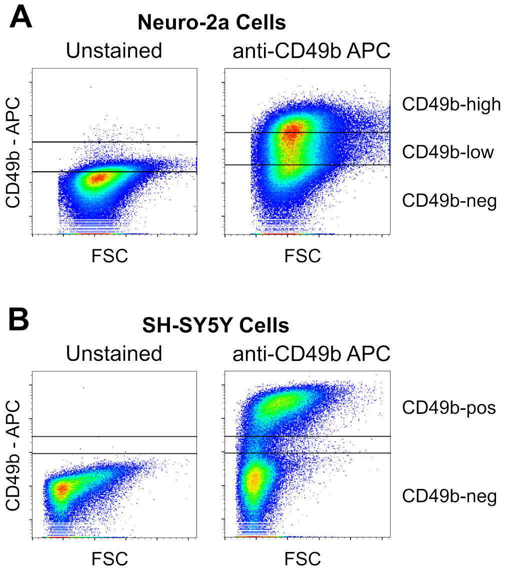

Fig. S1. Gating strategies for identifying CD49b populations

A) Representative examples of unstained (left) and CD49b-stained (right) N2a cells, with the gates used to identify CD49b-neg, CD49b-low, and CD49b-high cells.

B) Representative examples of unstained (left) and CD49b-stained (right) SH-SY5Y cells, with the gates used to identify CD49b-neg and CD49b-pos cells.

Disease Models & Mechanisms • Supplementary information

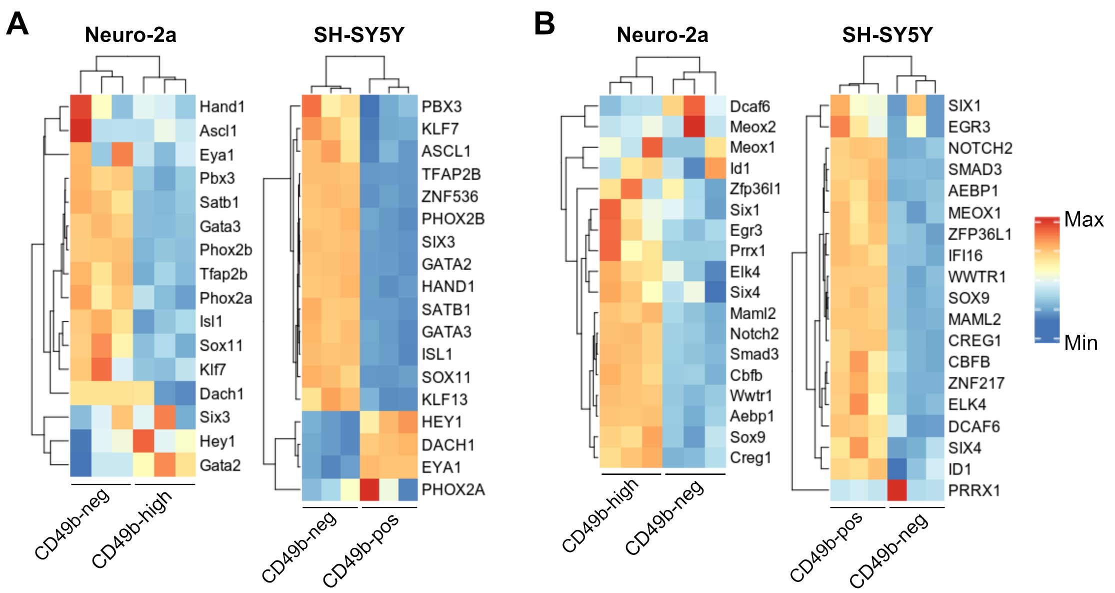

Fig. S2. Expression of transcription factor genes associated with adrenergic and mesenchymal neuroblastoma cell states, as described by van Groningen et al (2017)

A) Heatmaps showing expression of genes encoding for transcription factors associated with the adrenergic neuroblastoma cell state, showing enrichment in CD49b-neg cells in the indicated cell lines.
A) Heatmaps showing expression of genes encoding for transcription factors associated with the mesenchymal neuroblastoma cell state, showing enrichment in CD49b-pos/high cells in the indicated cell lines.

Disease Models & Mechanisms • Supplementary information

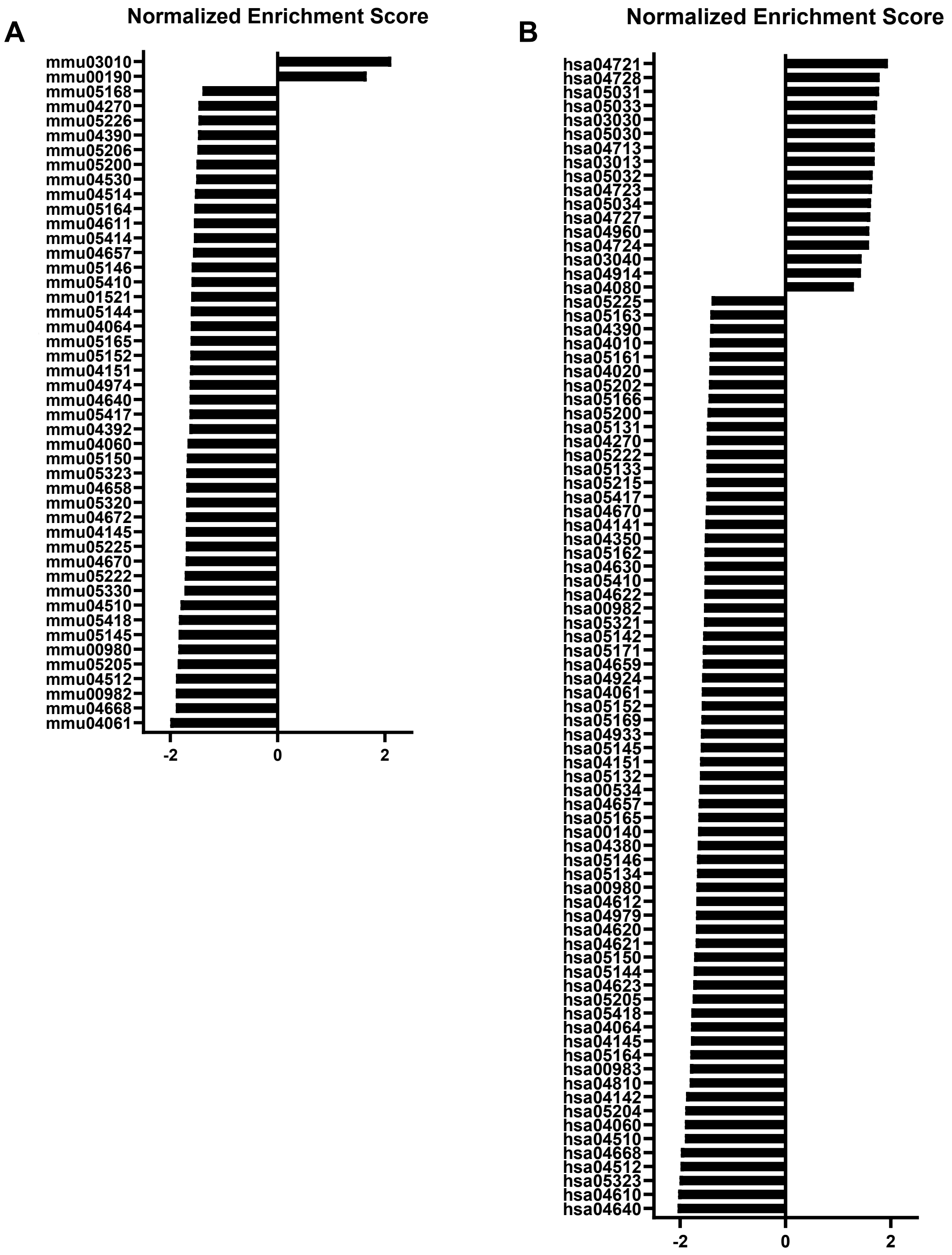

Fig. S3. KEGG pathways enriched in N2a cell populations

A) Normalized enrichment scores for KEGG pathways with adjusted p-value < 0.05 for CD49b-neg versus CD49b-high N2a cells. Scores < 0 indicate enrichment in CD49b-neg cells, while scores > 0 indicate enrichment in CD49b-high cells.

B) Normalized enrichment scores for KEGG pathways with adjusted p-value < 0.05 for CD49b-neg versus CD49b-pos SH-SY5Y cells. Scores < 0 indicate enrichment in CD49b-neg cells, while scores > 0 indicate enrichment in CD49b-pos cells.

Disease Models & Mechanisms • Supplementary information

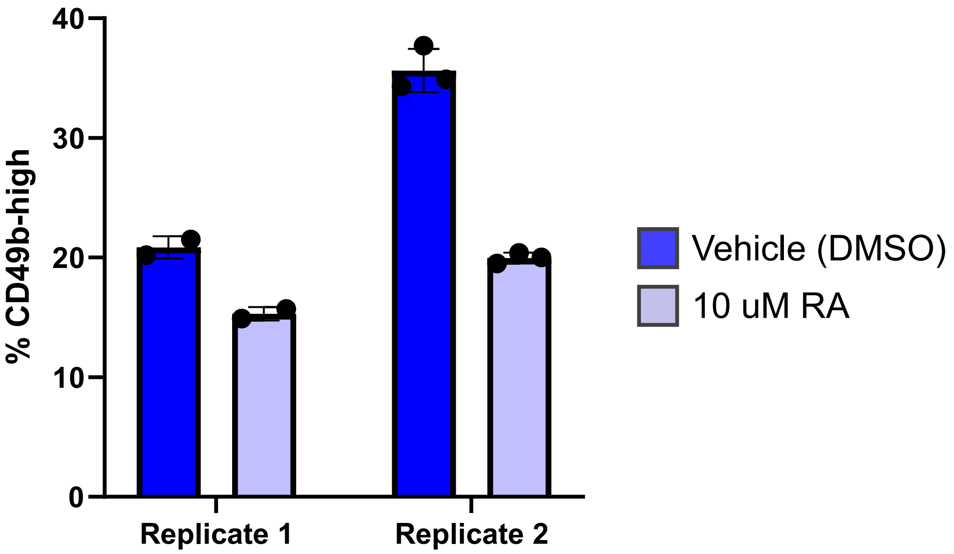

Fig. S4. RA treatment inhibits reversion to an immature cell state Quantification of the proportion of N2a cells in each CD49b expression category 7 days after sorting for CD49b-neg cells. n=2 for Replicate 1, n=3 for Replicate 2. Bars indicate mean, dots indicate individual replicates, and error bars indicate standard deviation. p=0.02 for Replicate 1, p=0.003 for Replicate 2.

Table S1. qPCR primer sequences

Mouse Primers

GENE	FORWARD PRIMER	REVERSE PRIMER
Gapdh	AGGTCGGTGTGAACGGATTTG	TGTAGACCATGTAGTTGAGGTCA
Elavl4	GCCTCAGGTGTCAAATGGACC	ACCCTAAACTCTGTCCTGTGAT
Phox2a	CTGGAGGCTTCCAATACAGTCC	GGAACTGCCGAGTAGGGTG
Phox2b	GGGCTAAGTTTCGCAAGCAG	CAGTGCTGTCGGGATCAGTG
Snap25	CAACTGGAACGCATTGAGGAA	GGCCACTACTCCATCCTGATTAT
Actl6b	GCGCTCGTCTTTGACATTGG	ATTGGTGTCGATGTGGAAAATCT
Itga2	TGTCTGGCGTATAATGTTGGC	CTTGTGGGTTCGTAAGCTGCT
Ngfr	CCTGGACAGTGTTACGTTCTC	ACACAGGGAGCGGACATACT

Human Primers

GENE	FORWARD PRIMER	REVERSE PRIMER
GAPDH	GGAGCGAGATCCCTCCAAAAT	GGCTGTTGTCATACTTCTCATGG
ELAVL4	AACCTCTATGTTAGCGGCCTT	TGGACACTCCTGTGACTTGAT
PHOX2A	GCGTTTTCGCTGAGACCCACTA	CGCTCCTGTTTGCGGAACTTGG
PHOX2B	AACCCGATAAGGACCACTTTTG	AGAGTTTGTAAGGAACTGCGG
SNAP25	ACCAGTTGGCTGATGAGTCG	CAAAGTCCTGATACCAGCATCTT
ACTL6B	ATCACACCTACAGCAAACACG	ACGTAGCCGTCATGTACTGGA
TUBB3	GGCCAAGGGTCACTACACG	GCAGTCGCAGTTTTCACACTC

Disease Models & Mechanisms • Supplementary information

Table S2. GSEA pathway enrichment in N2a CD49b-neg cells relative to CD49b-high cells.

	ID	Description	setSize	NES	pvalue	p.adjust
mmu05200	mmu05200	Pathways in cancer	420	-1.51265672622336	0.001024590163934	0.018976791896397
mmu05165	mmu05165	Human papillomavirus infection	290	-1.62157538065646	0.001046025104603	0.018976791896397
mmu04151	mmu04151	PI3K-Akt signaling pathway	268	-1.6302798263096	0.001061571125265	0.018976791896397
mmu04060	mmu04060	Cytokine-cytokine receptor interaction	168	-1.67946863491816	0.001121076233184	0.018976791896397
mmu05205	mmu05205	Proteoglycans in cancer	170	-1.86290899440938	0.001121076233184	0.018976791896397
mmu05417	mmu05417	Lipid and atherosclerosis	166	-1.64017792358256	0.001129943502825	0.018976791896397
mmu04510	mmu04510	Focal adhesion	165	-1.8086716535444	0.001131221719457	0.018976791896397
mmu05225	mmu05225	Hepatocellular carcinoma	144	-1.71038334206534	0.001152073732719	0.018976791896397
mmu04145	mmu04145	Phagosome	125	-1.71016350951094	0.001168224299065	0.018976791896397
mmu05152	mmu05152	Tuberculosis	126	-1.62249246313891	0.001168224299065	0.018976791896397
mmu05418	mmu05418	Fluid shear stress and atherosclerosis	112	-1.83804781328337	0.001177856301531	0.018976791896397
mmu05145	mmu05145	Toxoplasmosis	85	-1.84549176923768	0.001231527093596	0.018976791896397
mmu04670	mmu04670	Leukocyte transendothelial migration	87	-1.71088654107135	0.00123609394314	0.018976791896397
mmu04668	mmu04668	TNF signaling pathway	89	-1.89746165439036	0.001237623762376	0.018976791896397
mmu04974	mmu04974	Protein digestion and absorption	81	-1.63818712688457	0.001248439450687	0.018976791896397
mmu05222	mmu05222	Small cell lung cancer	76	-1.73086031452	0.00125313283208	0.018976791896397
mmu04512	mmu04512	ECM-receptor interaction	71	-1.89384289375118	0.001259445843829	0.018976791896397
mmu04061	mmu04061	Viral protein interaction with cytokine and cytokine recepto	48	-1.99724003152832	0.001335113484646	0.018976791896397
mmu00982	mmu00982	Drug metabolism - cytochrome P450	40	-1.89626897350245	0.001367989056088	0.018976791896397
mmu00980	mmu00980	Metabolism of xenobiotics by cytochrome P450	39	-1.85020160533763	0.001375515818432	0.018976791896397
mmu05320	mmu05320	Autoimmune thyroid disease	39	-1.70184648618894	0.001375515818432	0.018976791896397
mmu05330	mmu05330	Allograft rejection	34	-1.73587383931537	0.001410437235543	0.018976791896397
mmu05168	mmu05168	Herpes simplex virus 1 infection	343	-1.40074476604642	0.002061855670103	0.02653518601524
mmu04530	mmu04530	Tight junction	141	-1.51581663822736	0.002322880371661	0.028648857917151
mmu04658	mmu04658	Th1 and Th2 cell differentiation	70	-1.6998368143537	0.002522068095839	0.029861286254729
mmu04672	mmu04672	Intestinal immune network for IgA production	28	-1.70461075155689	0.002932551319648	0.03338596886984
mmu04514	mmu04514	Cell adhesion molecules	119	-1.53819344182852	0.0034924330617	0.036168132942327
mmu05164	mmu05164	Influenza A	116	-1.55209978304145	0.003504672897196	0.036168132942327
mmu05410	mmu05410	Hypertrophic cardiomyopathy	85	-1.60527293447506	0.003694581280788	0.036168132942327
mmu01521	mmu01521	EGFR tyrosine kinase inhibitor resistance	65	-1.61199366647466	0.003856041131105	0.036168132942327
mmu05323	mmu05323	Rheumatoid arthritis	63	-1.69773307475077	0.003896103896104	0.036168132942327
mmu04640	mmu04640	Hematopoietic cell lineage	58	-1.63934393741344	0.003916449086162	0.036168132942327
mmu05150	mmu05150	Staphylococcus aureus infection	42	-1.6879996836962	0.004032258064516	0.036168132942327
mmu05144	mmu05144	Malaria	35	-1.61683005999548	0.004178272980501	0.036375553006718
mmu05206	mmu05206	MicroRNAs in cancer	134	-1.49514463752825	0.004672897196262	0.037326607818411
mmu04390	mmu04390	Hippo signaling pathway	132	-1.48095958681023	0.004694835680751	0.037326607818411
mmu04611	mmu04611	Platelet activation	100	-1.56046082170593	0.004836759371221	0.037326607818411
mmu05414	mmu05414	Dilated cardiomyopathy	84	-1.5605553407886	0.004944375772559	0.037326607818411
mmu05146	mmu05146	Amoebiasis	76	-1.59972010293318	0.005012531328321	0.037326607818411
mmu04064	mmu04064	NF-kappa B signaling pathway	70	-1.61736386899207	0.005044136191677	0.037326607818411
mmu03010	mmu03010	Ribosome	111	2.12141168049714	0.006493506493506	0.046879949318974
mmu00190	mmu00190	Oxidative phosphorylation	122	1.65996330671413	0.006896551724138	0.047654824514329
mmu05226	mmu05226	Gastric cancer	122	-1.47475851509619	0.007001166861144	0.047654824514329
mmu04270	mmu04270	Vascular smooth muscle contraction	113	-1.47420278440162	0.007083825265643	0.047654824514329
mmu04657	mmu04657	IL-17 signaling pathway	72	-1.57621214771262	0.007575757575758	0.049045599151644
mmu04392	mmu04392	Hippo signaling pathway - multiple species	23	-1.64595195248203	0.007621951219512	0.049045599151644

Disease Models & Mechanisms • Supplementary information

Table S3. GSEA pathway enrichment in SH-SY5Y CD49b-neg cells relative to CD49b-pos cells.

	ID	Description	setSize	NES	pvalue	p.adjust
hsa05200	hsa05200	Pathways in cancer	473	-1.48330639545081	0.001058201058201	0.018417874396135
hsa04151	hsa04151	PI3K-Akt signaling pathway	311	-1.62216832749039	0.001095290251917	0.018417874396135
hsa05165	hsa05165	Human papillomavirus infection	300	-1.6541949328083	0.001097694840834	0.018417874396135
hsa05132	hsa05132	Salmonella infection	238	-1.62419871523442	0.001129943502825	0.018417874396135
hsa04510	hsa04510	Focal adhesion	188	-1.90914309938738	0.001166861143524	0.018417874396135
hsa05205	hsa05205	Proteoglycans in cancer	189	-1.76514933838381	0.001169590643275	0.018417874396135
hsa04060	hsa04060	Cytokine-cytokine receptor interaction	199	-1.90838261245387	0.001170960187354	0.018417874396135
hsa04810	hsa04810	Regulation of actin cytoskeleton	201	-1.81756072554295	0.001172332942556	0.018417874396135
hsa05171	hsa05171	Coronavirus disease - COVID-19	192	-1.56829203825532	0.001173708920188	0.018417874396135
hsa04141	hsa04141	Protein processing in endoplasmic reticulum	163	-1.52138479407604	0.001177856301531	0.018417874396135
hsa05169	hsa05169	Epstein-Barr virus infection	174	-1.5961763806023	0.001183431952663	0.018417874396135
hsa04621	hsa04621	NOD-like receptor signaling pathway	148	-1.7046435313282	0.001200480192077	0.018417874396135
hsa05164	hsa05164	Influenza A	141	-1.80661479075298	0.001212121212121	0.018417874396135
hsa05418	hsa05418	Fluid shear stress and atherosclerosis	125	-1.78988631541278	0.00121802679659	0.018417874396135
hsa04142	hsa04142	Lysosome	122	-1.88707940716617	0.001219512195122	0.018417874396135
hsa04145	hsa04145	Phagosome	131	-1.79573677217809	0.001228501228501	0.018417874396135
hsa04668	hsa04668	TNF signaling pathway	97	-1.98616224016665	0.001280409731114	0.018417874396135
hsa04512	hsa04512	ECM-receptor interaction	82	-1.99430708198868	0.001295336787565	0.018417874396135
hsa04640	hsa04640	Hematopoietic cell lineage	70	-2.04926388367884	0.001335113484646	0.018417874396135
hsa05323	hsa05323	Rheumatoid arthritis	68	-2.01318188245225	0.001353179972936	0.018417874396135
hsa00983	hsa00983	Drug metabolism - other enzymes	62	-1.81605966112051	0.001375515818432	0.018417874396135
hsa04610	hsa04610	Complement and coagulation cascades	56	-2.03648423788626	0.001381215469613	0.018417874396135
hsa05204	hsa05204	Chemical carcinogenesis - DNA adducts	45	-1.90333770624806	0.001410437235543	0.018417874396135
hsa04623	hsa04623	Cytosolic DNA-sensing pathway	42	-1.75365611198774	0.001449275362319	0.018417874396135
hsa05131	hsa05131	Shigellosis	237	-1.49479145987255	0.00225988700565	0.022060684966186
hsa05417	hsa05417	Lipid and atherosclerosis	176	-1.50180125493819	0.002364066193853	0.022060684966186
hsa05152	hsa05152	Tuberculosis	139	-1.59065286427773	0.002433090024331	0.022060684966186
hsa04630	hsa04630	JAK-STAT signaling pathway	132	-1.53707873823574	0.002457002457002	0.022060684966186
hsa04380	hsa04380	Osteoclast differentiation	112	-1.66925944750076	0.002493765586035	0.022060684966186
hsa04064	hsa04064	NF-kappa B signaling pathway	90	-1.79156252917088	0.002564102564103	0.022060684966186
hsa04933	hsa04933	AGE-RAGE signaling pathway in diabetic complications	94	-1.60506019889218	0.002567394094994	0.022060684966186
hsa05145	hsa05145	Toxoplasmosis	95	-1.60909534308286	0.002587322121604	0.022060684966186
hsa05146	hsa05146	Amoebiasis	82	-1.67885526334564	0.00259067357513	0.022060684966186
hsa04620	hsa04620	Toll-like receptor signaling pathway	81	-1.70201820754432	0.002604166666667	0.022060684966186
hsa04612	hsa04612	Antigen processing and presentation	53	-1.69288962908142	0.002770083102493	0.022060684966186
hsa00980	hsa00980	Metabolism of xenobiotics by cytochrome P450	52	-1.68975130378983	0.002777777777778	0.022060684966186
hsa05134	hsa05134	Legionellosis	49	-1.68109168873551	0.00278940027894	0.022060684966186
hsa04979	hsa04979	Cholesterol metabolism	45	-1.69646482626863	0.002820874471086	0.022060684966186
hsa05150	hsa05150	Staphylococcus aureus infection	45	-1.73337729528514	0.002820874471086	0.022060684966186
hsa05144	hsa05144	Malaria	37	-1.74706018587023	0.002967359050445	0.022139953542393
hsa05033	hsa05033	Nicotine addiction	31	1.74099699858358	0.00297619047619	0.022139953542393
hsa05030	hsa05030	Cocaine addiction	44	1.69969776807061	0.003311258278146	0.023696068783343
hsa04010	hsa04010	MAPK signaling pathway	275	-1.43872824449531	0.003340757238307	0.023696068783343
hsa05031	hsa05031	Amphetamine addiction	61	1.77858553808803	0.003571428571429	0.024756493506494
hsa04657	hsa04657	IL-17 signaling pathway	73	-1.64797255870272	0.003896103896104	0.026365836791148
hsa04061	hsa04061	Viral protein interaction with cytokine and cytokine receptoı	57	-1.58956917227691	0.004120879120879	0.026365836791148
hsa04924	hsa04924	Renin secretion	55	-1.58544813336996	0.004132231404959	0.026365836791148
hsa04721	hsa04721	Synaptic vesicle cycle	72	1.94541428715597	0.004149377593361	0.026365836791148

Disease Models & Mechanisms • Supplementary information

hsa05032	hsa05032	Morphine addiction	80	1.65916238276321	0.004273504273504	0.026600383743241
hsa05166	hsa05166	Human T-cell leukemia virus 1 infection	206	-1.46091051179936	0.004645760743322	0.026810829817159
hsa04020	hsa04020	Calcium signaling pathway	211	-1.44437129201528	0.004667444574096	0.026810829817159
hsa04713	hsa04713	Circadian entrainment	89	1.69484488381269	0.004694835680751	0.026810829817159
hsa05202	hsa05202	Transcriptional misregulation in cancer	161	-1.45250153901092	0.004711425206125	0.026810829817159
hsa00534	hsa00534	Glycosaminoglycan biosynthesis - heparan sulfate / hepari	21	-1.63489955335192	0.004746835443038	0.026810829817159
hsa03013	hsa03013	Nucleocytoplasmic transport	103	1.69410186314273	0.004878048780488	0.02688883011549
hsa05162	hsa05162	Measles	116	-1.53542495019082	0.004956629491945	0.02688883011549
hsa04724	hsa04724	Glutamatergic synapse	106	1.58963459684226	0.005025125628141	0.02688883011549
hsa04728	hsa04728	Dopaminergic synapse	124	1.78958952272942	0.005376344086022	0.028272154245458
hsa04723	hsa04723	Retrograde endocannabinoid signaling	138	1.64145538585135	0.005649717514124	0.029206166810304
hsa05163	hsa05163	Human cytomegalovirus infection	192	-1.42762080633599	0.005868544600939	0.029322411936625
hsa04270	hsa04270	Vascular smooth muscle contraction	119	-1.49599962429823	0.006142506142506	0.029322411936625
hsa05034	hsa05034	Alcoholism	166	1.62493911143977	0.006289308176101	0.029322411936625
hsa03030	hsa03030	DNA replication	36	1.70049898580137	0.006309148264984	0.029322411936625
hsa04350	hsa04350	TGF-beta signaling pathway	89	-1.53251681343186	0.006337135614702	0.029322411936625
hsa05142	hsa05142	Chagas disease	87	-1.56235404024528	0.006337135614702	0.029322411936625
hsa04659	hsa04659	Th17 cell differentiation	88	-1.56910059359987	0.006345177664975	0.029322411936625
hsa05410	hsa05410	Hypertrophic cardiomyopathy	85	-1.53823037081303	0.006443298969072	0.029331435605478
hsa04622	hsa04622	RIG-I-like receptor signaling pathway	55	-1.54107614983334	0.006887052341598	0.030890455355696
hsa05161	hsa05161	Hepatitis B	145	-1.4436785971325	0.007185628742515	0.031762561832856
hsa04670	hsa04670	Leukocyte transendothelial migration	103	-1.51475911222153	0.007528230865747	0.03280157734361
hsa05215	hsa05215	Prostate cancer	92	-1.50148611373411	0.007692307692308	0.033044420368364
hsa00140	hsa00140	Steroid hormone biosynthesis	39	-1.66047620328078	0.008759124087591	0.037104622871046
hsa04080	hsa04080	Neuroactive ligand-receptor interaction	264	1.30013987928096	0.009090909090909	0.037982565379826
hsa04960	hsa04960	Aldosterone-regulated sodium reabsorption	36	1.59172373533591	0.009463722397476	0.039005882854463
hsa00982	hsa00982	Drug metabolism - cytochrome P450	48	-1.54613559181687	0.00974930362117	0.039180063128533
hsa05321	hsa05321	Inflammatory bowel disease	50	-1.54756824297772	0.00976290097629	0.039180063128533
hsa05222	hsa05222	Small cell lung cancer	87	-1.49862413342115	0.010139416983523	0.040162625713957
hsa05225	hsa05225	Hepatocellular carcinoma	157	-1.40502785949715	0.010701545778835	0.041606984516745
hsa03040	hsa03040	Spliceosome	126	1.44715352337382	0.010869565217391	0.041606984516745
hsa04390	hsa04390	Hippo signaling pathway	140	-1.42929260579727	0.010962241169306	0.041606984516745
hsa05133	hsa05133	Pertussis	60	-1.50132128899966	0.011049723756906	0.041606984516745
hsa04727	hsa04727	GABAergic synapse	81	1.61348086512485	0.012820512820513	0.047686053783615
hsa04914	hsa04914	Progesterone-mediated oocyte maturation	94	1.43514554917613	0.013452914798206	0.049435409800638

Disease Models & Mechanisms • Supplementary information
